# Dissemination Dynamics of Receding Words: A Diachronic Case Study of *Whom*

**DOI:** 10.3389/frai.2021.654154

**Published:** 2021-06-29

**Authors:** Axel Bohmann, Martin Bohmann, Lars Hinrichs

**Affiliations:** ^1^ Englisches Seminar, Albert-Ludwigs-Universität Freiburg, Freiburg, Germany; ^2^ Institute for Quantum Optics and Quantum Information - Vienna (IQOQI), Austrian Academy of Sciences, Vienna, Austria; ^3^ Vienna Center for Quantum Science and Technology (VCQ), Vienna, Austria; ^4^ Department of English, The University of Texas at Austin, Austin, TX, United States

**Keywords:** dissemination, sociolinguistics, receding features, whom, relativizers, register

## Abstract

We explore the relationship between word dissemination and frequency change for a rapidly receding feature, the relativizer *whom*. The success of newly emerging words has been shown to correlate with high dissemination scores. However, the reverse—a correlation of lower dissemination scores with receding features—has not been investigated. Based on two established and two newly developed measures of word dissemination—across texts, linguistic environments, registers, and topics—we show that a general correlation between dissemination and frequency does not obtain in the case of *whom*. Different dissemination measures diverge from each other and show internally variable developments. These can, however, be explained with reference to the specific sociolinguistic history of *whom* over the past 300 years. Our findings suggest that the relationship between dissemination and word success is not static, but needs to be contextualized against different stages in individual words’ life-cycles. Our study demonstrates the applicability of large-scale, quantitative measures to qualitatively informed sociolinguistic research.

## Introduction

### The Sociolinguistics of Emergence and Attrition

Sociolinguistic research is predominantly concerned with the emergence and spread of linguistic innovations, but has paid less attention to the dynamics of receding features. The canonical S-curve pattern of linguistic change (Labov, 1994) proceeds along three idealized stages—barely perceptible incipient change, rapid frequency increase through incrementation, and establishment of the feature within the community—to a theoretical steady state. Feature dynamics beyond this point are less well-understood. Yet, sociolinguists stand to gain insight from attention to receding features. These are of interest in their own right as part of a community’s repertoire, but also because systematic comparison of the dynamics involved in feature emergence and attrition can lead to a more comprehensive understanding of linguistic change in general.

The dynamics of lexical emergence have recently been addressed through large-scale computational-statistical methods. Grieve et al. (2017) develop a procedure to identify emerging words in a corpus of 8.9 billion Twitter messages, based on initially low frequency and a high increase in frequency over a given time period. In a follow-up study, Grieve (2018) predicts the further success of 54 emerging words identified in Grieve et al. (2017) as a function of word length, part-of-speech, underlying word-formation process, and novelty of the word’s referent. The latter predictor is shown to be particularly relevant in determining the frequency development of innovative words, whereas part-of-speech does not appear to play a significant role.

A further important predictor of a word’s success is its social dissemination, defined by Altmann et al. (2011) as the ratio between the number of social units (e.g. speakers or texts) in a sample that use the word and the expected number of social units using the word. This expected number is calculated under the assumption of random spread of the word across social units, given its relative frequency and each social unit’s total word count. Altmann et al. (2011) and Altmann et al. (2013) find higher dissemination scores to be a strong predictor of a word’s continued increase in frequency.

The notion of social dissemination has been taken up in Garley and Hockenmaier (2012) as well as in Stewart and Eisenstein (2018). In both of these studies, its predictive power is less evident, which may in part be attributed to the inclusion of proper nouns in Altmann et al. (2011). Usage of these may be more directly linked to social dynamics than usage of general innovations (Stewart and Eisenstein, 2018: 4368). Stewart and Eisenstein extend the concept of dissemination from the social to the linguistic context of words. They calculate linguistic dissemination based on a comparison between expected and observed unique trigram frequencies in which a given word occurs and show, on the basis of several statistical models, that this metric effectively predicts future frequency developments.

These large-scale, quantitative findings are conceptually related to recent work in a more qualitative perspective. Squires (2014) traces how one specific phrase coined by a TV personality is taken up on Twitter. After being used by fans of the show the phrase originates from in direct reference to the initial situation of utterance, the phrase gradually spreads to wider discursive contexts and becomes increasingly detached from its origin. Squires (2014) refers to this process as “indexical bleaching.” Given that indexicality describes the connection of a sign to the specific contexts it is embedded in, the notion of indexical bleaching may be related to Altmann et al.’s (2011) concept of social dissemination, including its extension in Stewart and Eisenstein (2018): the further a linguistic unit is indexically bleached, the more evenly disseminated it can be expected to be. One important thing to note about Squires’ research is that her focusing on an individual form allows her to trace in more detail the indexical dynamics involved in its spread. As such, her analysis is able to go beyond a static relationship between indexical focus and a word’s successful spread. She concludes that “indexical strength catalyzes uptake, but indexical loss facilitates diffusion” (Squires, 2014: 58).

This observation implies that the role of dissemination (which we take to be inversely related to indexical strength) in predicting a form’s future frequency development may assume different shapes at different stages of that form’s life-cycle. Most of the studies cited above have restricted their focus to the rapid emergence of innovative words, and to predictions about their relatively short-term success. Altmann et al. (2013) also consider the development of established words over longer time periods, yet their focus remains on frequency increase. The extent to which the dynamics of receding forms, i.e. those that are firmly established in the language but decrease in frequency, mirrors those of emerging ones is currently not well understood.

We focus on one particular such form, the relativizer *whom*, in order to shed light on the question of how frequency decline interacts with dissemination during an extended phase of attrition. In addition to implementing Altmann et al.’s (2011) original measure and Stewart and Eisenstein’s (2018) extension of it, we also address dissemination across registers and topics. This is done on the basis of a multi-dimensional analysis (Biber, 1988) and a topic model for the corpus under consideration. In contrast to Altmann et al.’s (2011) approach, focusing on text-level properties like register and topic enables us to treat the range of texts in our corpus not simply as distinct units, but to systematically relate them to one another in terms of their linguistic characteristics and discourse content. Tracing the association between a form and specific register contexts and topics is arguably a more immediate window into indexical focusing than simply quantifying its presence or absence in a number of texts which are conceived as otherwise undifferentiated units. Compared to Stewart and Eisenstein’s (2018) measure, our newly developed dissemination indices relate to characteristics of the textual environment on the whole, instead of the immediate collocation behavior of a word.

### A Rapidly Receding Word: *Whom*

Standard English allows for nine different devices to introduce relative clauses (RCs): *that, which, who, whose, whom, when, where, why*, and zero (that is, the absence of an overt element introducing a relative clause). Competition among these forms is in part governed by categorical rules, e.g. the fact that *that* is only permissible for introducing restrictive RCs, and in part by probabilistic constraints. The latter have been the focus of many recent studies and are relatively well-documented for the three most prolific members of the set, *which*, *that*, and *zero* (e.g. Guy and Bayley, 1995; Levey, 2006; Hinrichs et al., 2015). In addition to language-internal constraints like antecedent noun phrase length, RC length and whether the relativizer assumes the subject or object role in the RC, Hinrichs et al. (2015) show that relativizer choice is susceptible to the influence of prescriptivist norms. Together with broader stylistic drifts, such as the colloquialization of written English (Leech et al., 2009), these factors account for a marked frequency decrease of *which* during the second half of the 20th century.

Although characterized by a similarly drastic decline in frequency over the past 200 years, *whom* has received comparatively less attention. This form is commonly regarded as a case-marked variant of *who* expressing objective case, analogous to the correspondence between *she* and *her*, *he* and *him,* etc. (although see Lasnik and Sobin (2000) for a competing account). Accordingly, traditional prescriptive grammar would require *whom* instead of *who* in RCs with human antecedents in which the relativizer occurs in the object position (Aarts, 1994: 73), as in (1).

(1) going for the jugular of anyone **whom** he considers an enemy <COHA_fic_1988_782035>

As early as 1921, Sapir (1921: 167) predicted that “within a couple of hundred years from to-day not even the most learned jurist will be saying ‘Whom did you see?’” Sapir identified several factors that conspire to render *whom* a moribund form: the general erosion of the English case-inflectional paradigm, the isolation of *whom* from the case-invariant remaining relativizers on the one hand and the system of personal pronouns on the other, as well as a purported “clumsiness” (Sapir, 1921: 171) in its phonetic shape. He further anticipated a general retreat of *who* and its variants from the class of relativizers in favor of highlighting their role as interrogative pronouns.

**FIGURE 1:**
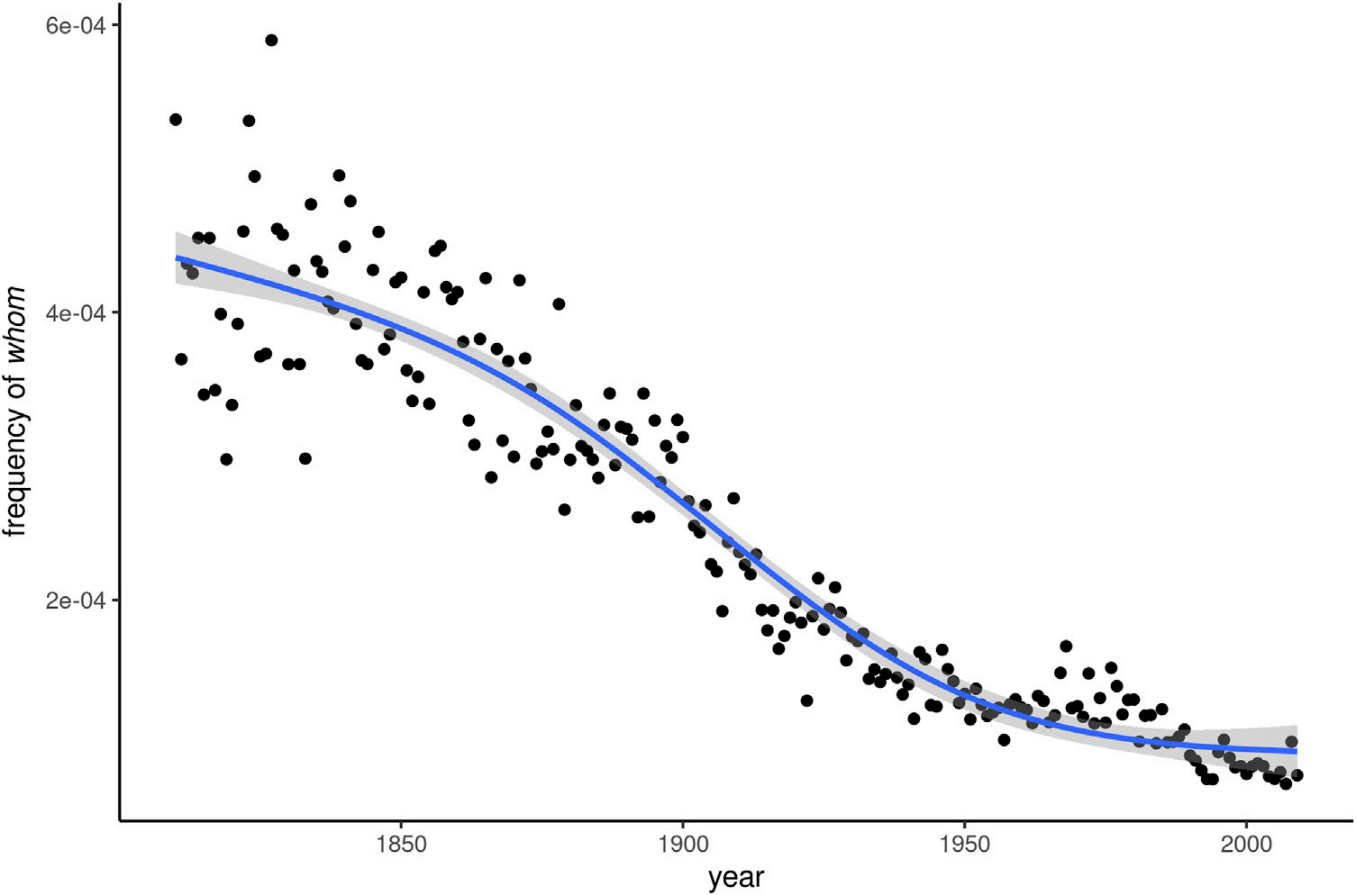
Frequency development of *whom* over 180 years of written American English.

Many of these predictions have been borne out over the past 100 years. In the Corpus of Historical American English (COHA; Davies, 2010), the relative frequency of *whom* is consistently about an order of magnitude smaller than that of *who* throughout the 20th century. In terms of relative frequency, COHA shows a steady decrease of *whom* between 1810 and 2009, as can be seen in Figure 1. Using the Spearman correlation coefficient between relative frequency and year as an operationalization of the rise or fall of a word, *whom* is the fifth most rapidly receding item in the entire corpus, after *shall*, *nor*, *vain*, and *whence*. Along with this general decline, linguists have noted an increasing stylistic restriction to formal contexts, with prescriptivist discourse as an important catalyst (Aarts, 1994).

Figure 1, however, also shows that the rate of decline has slowed considerably in the second half of the 20th century. Empirical research on the recent past of written English has come to varied conclusions as to the fate of *whom*. Bauer (1994: 76) contends that avoidance of the word “has probably been noticeable throughout the [20th] century” and that ongoing change is relatively negligible, an observation also shared by Mair (2006: 141–143). Aarts and Aarts (2002: 128), on the other hand, find “staggering” rates of decline, both in written and spoken corpora, between the 1960s and the 1990s. Figure 1, based on larger and more systematic corpus data than the studies previously cited, would seem to confirm Mair (2006: 143) verdict that “*whom* now seems to have reached the tail end of the characteristic S-shaped curve of progression in linguistic change.”

Despite disagreement about the most recent frequency developments, there is overwhelming consensus in the literature regarding the stylistic aspects of *whom,* namely: a strong association with very formal, almost exclusively written kinds of discourse. The fact that *whom* has not completely disappeared from the language is often attributed to its institutional backing in the educational system (Mair, 2006: 134). A discrepancy between actual usage and prescriptive norms means that most people “will recognize it as correct in a wider range of contexts […], but probably not use it” (Bauer, 1994: 77).

The strong stylistic connotations of *whom* are evident in meta-linguistic discourse as well. In present-day internet culture, a class of memes is circulating which capitalizes on these indexicalities. The structural template for these memes pairs a sequence of images with a sequence of words. The images are repetitions of the same motif, a stylized X-ray of a human head, showing a rise in brain size with every iteration. The words form the sequence *who—whom—whoms—whomst’d*.^1^ The rhetorical effect is an equation of linguistic forms with levels of intellectual superiority. The fact that both *whomst* and *whomst’d* are nonce words created for the context of this meme indicates the level of metalinguistic play inherent in it. These two words are constructed by attaching graphemic material to the base word that does not add any semantic content. In the case of *whomst*, it is likely that the -*st* sequence is used in analogy to archaic second-person singular verb inflections that were still common in Early Modern English. The grammatical information these suffixes used to bear is nowadays encoded on the subject only. The position of *whom* in this sequence construes this form as similarly burdened by unnecessary graphemic material but indicative of intellectual attainment. The meme consequently suggests a change in status for *whom* in that it has largely lost its grammatical function of case-distinction but gained indexical strength linking it to educated and hyper-formal contexts.

The properties described above make *whom* a suitable candidate for a contextualized analysis of various dissemination measures. Its frequency development over the past 200 years follows a clear trajectory which mirrors that of the S-curve often observed in the spread of linguistic innovations. The factors contributing to its decline, while not yet analyzed in a quantitative perspective, are well-attested. In addition, metalinguistic discourse surrounding the correct usage of *whom* in the form of prescriptive and descriptive linguists’ comments is documented for at least as far back as the 18th century (Aarts, 1994). These facts enable us to formulate specific hypotheses regarding the dissemination of *whom* at different time periods and to contextualize observable dissemination developments against prior knowledge about the feature.

### Research Objectives

We investigate the dynamics of dissemination that *whom* has undergone over the course of 180 years, between 1830 and 2009. Based on four quantitative measures, two established and two newly developed ones, we trace change in the dissemination of *whom* in this time period, which is characterized by continuous, but abating frequency decline. As can be seen in Figure 1, this decline is particularly rapid in the second half of the 19th and the first half of the 20th century, with the slope flattening again after around 1950.

On the basis of the literature on success during emergence, summarized in *The Sociolinguistics of Emergence and Attrition*, it would be valid to expect decrease in frequency to correspond with decrease in dissemination. This is the general statistical relationship that obtains in all the quantitative studies cited above, and is also a plausible hypothesis on purely theoretical terms. In the power-law distribution of any language’s vocabulary, the most common items are likely shared by all speakers and across contexts, whereas low-frequency items in the long tail of the distribution can be expected to show stronger contextual sensitivity (Kretzschmar, 2015), i.e. lower dissemination. As a word’s general frequency declines, one may consequently expect it to specialize into narrower niches of usage. In analogy to Squires’ (2014) term, we call this process “indexical focusing.” The tendency of receding forms to cluster in formulaic expressions serves as a case in point. In its extreme version, this process leaves receding words entirely unproductive and semantically intransparent outside of the larger constructions they are embedded in. Examples of such items include the highlighted words in the expressions *to make short shrift* or *kith and kin*. The baseline hypothesis for the analysis below, then, is that the frequency decline of *whom* will coincide with a decline in dissemination. However, Squires (2014) reminds us that this relationship may not be static.

Our focus on an individual word comes at the expense of generalizability. There is no guarantee that the dynamics we observe for *whom* are shared by all, or even the majority of, receding forms in the language. While recognizing this limitation, we suggest that this narrow focus also brings important advantages. In order to make statistical generalizations like those described in Altmann et al. (2011), Altmann et al. (2013), or Stewart and Eisenstein (2018) more immediately relevant to sociolinguistic research, they need to be understood in relation to individual features of interest. Unlike phenomena in statistical physics and other core sciences, words in a language are not merely units with certain statistical properties, but are embedded in individual histories of social meaning and metalinguistic reflection. The sociolinguistic record contains a large number of features about which a good deal is known in this respect. It is consequently possible to formulate specific expectations as to the relationship between frequency developments and dissemination measures for such features that go beyond general regularities. A consideration of individual words’ social role in conjunction with observable statistical properties promises to enrich our understanding of both these perspectives.

Our aim is to make the notion of dissemination tangible from a situated sociolinguistic perspective and to evaluate the utility of each dissemination measure for future application in contextualized sociolinguistic research. Specifically, we ask how well the four measures correlate with change in frequency, as well as how strongly correlated they are with each other. If no correlation between frequency and a given dissemination measure can be found, the utility of that measure is up to question. If the dissemination measures show no or only weak correlation amongst each other, this fact requires further attention. Our assumption is that, despite being operationalized at different levels, dissemination is a general property which we expect to take a similar shape independent of its precise quantification.

## Materials and Methods

### Corpus

Our analysis is based on the Corpus of Historical American English (COHA; Davies, 2010), which includes samples of written American English for each year between 1810 and 2009. The corpus is sub-divided into four genres: news, magazine, fiction, and non-fiction writing. Each word in the corpus is annotated with lemma and part-of-speech information.

Due to the difficulty of sampling historical language data, several aspects of the COHA sampling frame are not consistent throughout the 200 years it covers. For instance, the sparsity of texts for some genres from the more distant past has resulted in the inclusion of fewer, but longer individual texts for much of the 19th century. Further, newspaper texts are only sampled from 1860 onwards and different archives were used for the extraction of text samples for different time periods.^2^ The effect of archival sources is visible especially for magazine writing, for which our register analysis (see below) shows a marked difference between texts before and after 1900.

Consideration of the above factors led us to exclude the first two decades of COHA (1810–1829) from the analysis. With a median number of 14.5 texts per year, these do not offer sufficient data for our analyses, most of which treat individual texts as the relevant units. We further note that the irregularities mentioned above are not fully resolved before the sampling point 1925. From this time on, both the archives used for text sampling and the mean number and word count of texts per year are consistent. While our analysis covers the years from 1830 up to 2009, then, the results are expected to be most robust for the latter half of this time period.

We work with the full-text, offline version of COHA, which includes lemma and part-of-speech information for each word. For each year between 1830 and 2009, we calculate the four dissemination measures for *whom* described in the following sections.

### Social Dissemination

Following Altmann et al. (2011), we measure social dissemination (D^S^), as the ratio between the observed and expected social units a word occurs in at a given time. For our purposes, the social units of relevance are the individual corpus texts. In other words, we divide the number of documents *whom* occurs in by the number of documents it is expected to occur in. To calculate the latter number, a probability of observing *whom* in each text is calculated based on the text’s word count and the relative frequency of *whom* in the corpus at the time point under consideration. These probabilities are then summed to approximate the expected document count. The assumption for this baseline model is that all words occur randomly in the texts, with a probability corresponding to their relative frequency. The probability to find the word *whom* at least once in the *i* ^th^ text of word length $m_{i}$ is then given by $T_{i}=1-e^{-fm_{i}}$, where f is the relative frequency of *whom* in the considered year. Based on this, we can calculate the expected number texts containing *whom* via $\tilde{T}=\overset{N_{T}}{\underset{i=1}{\sum }}T_{i}$, where $N_{T}$ is the number of texts in the considered year. With this expectation of the baseline model, we can calculate the dissemination coefficient

$$D^{S}=\frac{T}{\tilde{T}}$$

which is the ratio between the number of texts in which *whom* is used T and the expected number of texts following the baseline model. A value of D^S^ = 1 corresponds to dissemination of a word across texts as if its occurrence was entirely random. Values below 1 indicate “clumping” (Altmann et al., 2013: 3), i.e. the use of the word in a smaller set of texts than expected. The closer to 0 D^S^ is, the less regularly disseminated the corresponding word is. Under-dissemination is interpreted by Altmann et al. (2013) as a sign of low word vitality.

### Linguistic Dissemination

Stewart and Eisenstein (2018) define linguistic dissemination (D^L^) as the difference between the log count of unique trigrams a word occurs in $\left(C^{3}\right)$ and the word’s expected log unique trigram count $\left({\tilde{C}}^{3}\right)$. Since the logarithms of frequency and unique trigram count are highly correlated (Egghe, 2007; Stewart and Eisenstein, 2018: 4364), it is possible to calculate the expected log trigram count based on a word’s frequency. In Stewart and Eisenstein (2018), this is done by fitting a linear model for all words at a given time point, with the words’ log frequencies as the predictor and their log trigram counts as the outcome variable. Linguistic dissemination is then defined as the residual error between the model prediction and the observed log trigram count $\left(D^{L}=C^{3}-{\tilde{C}}^{3}\right)$. Positive values indicate higher-than-expected numbers of trigrams, i.e. particular linguistic versatility, whereas negative values indicate a restriction of the linguistic contexts a word occurs in. Negative D^L^ is a predictor of frequency decline.

We treat individual sentences as the relevant context for trigram detection and do not consider trigrams across sentence boundaries. Each document in the raw, unannotated version of COHA is split at sentence-final punctuation marks (periods, question and exclamation marks, semicolons, and colons). For copyright reasons, the offline version of COHA replaces sequences of ten words at set intervals with ten *@* symbols. We treat these like sentence-final punctuation and do not allow trigrams to extend across them. If a word occurs in a place in the sentence that does not permit a right or a left trigram neighbor, i.e. in the first, second, last, or second-to-last position, we still register three unique trigrams. In these cases, we insert “<START>” or “<END>” instead of actual words into the trigram in order to replicate the method in Stewart and Eisenstein (2018).

Counting all trigrams for each word at a given time period proved computationally intractable. We therefore restrict ourselves to a random selection from a list of 17,912 words that occur at least 1,000 times in the corpus on the whole. For each time period, 10,000 items from this list of words are drawn and their unique trigram counts and frequencies of occurrence are measured. Given the regular relationship between log frequency and log unique trigram count, this amount of data is sufficient to reliably estimate the coefficient of the linear model and hence D^L^.

### Register Dissemination

In addition to social and linguistic dissemination, we also propose a measure of register dissemination (D^R^). Our notion of register is closely in line with that developed by Biber (e.g. Biber, 1988; Biber and Conrad, 2009; Biber, 2012), both in how we conceptualize and how we quantify it. The term is defined as “a variety associated with a particular situation of use” (Biber and Conrad, 2009: 6). While the relevant situational parameters may relate to medium and context of communication, communicative goals and norms, and a number of other extra-linguistic factors (see Biber and Conrad, 2009: chap. 2), they have a direct and measurable bearing on the linguistic properties of a stretch of discourse.

To measure the interrelationship between situational properties and linguistic characteristics, the exploratory method of multi-dimensional analysis (MDA; Biber, 1988) has been established in the corpus-linguistic community. This method proceeds by compiling a corpus of relevance for the analysis, i.e. one that represents the situational parameters of interest, as well as a number of linguistic features hypothesized to play an important role in register differentiation. Such features are usually relatively common, high- to mid-frequency ones, such as the frequency of passive-voice constructions, personal pronouns, or non-standard words in a text. For each corpus text, the frequency profile of each feature is measured. The resulting text-feature matrix is subjected to exploratory factor analysis (Thompson, 2004) in order to discover a small number of latent “dimensions of variation” (Biber, 1988) that capture a large amount of the total variance of the extracted features. Each dimension is characterized by the linguistic features it is most strongly associated with, and each corpus text can be scored on a continuum for each dimension. Qualitative consideration of the most strongly associated features and the highest- or lowest-scoring kinds of texts for a dimension drives the interpretation and labeling of each dimension.

We perform such an analysis for the entirety of the COHA data. We use 65 of the features proposed in Biber (1988) and 24 additional ones largely adapted from Bohmann (2019). In addition, we also include the relative frequency of each of the 100 most common part-of-speech trigrams in COHA. The resulting 116,614 × 179 text-feature matrix is subjected to factor analysis with the psych package (Revelle, 2020) in R (R Core Team, 2020). Following an inspection of the variances accounted for by the first 100 components of a principal component analysis over the features, we decided to extract five factors from the data. We use a principal axes factor solution rotated to the promax criterion, which allows for moderate inter-factor correlations. The factor scores for each text are calculated using the regression method (see Thompson, 2004; Revelle, 2020 for details).

Space does not permit a full discussion of the dimensions and the qualitative process that produced interpretations and labels for each. Here, we restrict ourselves to an overview in tabular form. Table 1 shows the five dimensions (i.e. factors developed in the factor analysis) with the labels we have chosen for them. The most strongly associated features, genres, and the dimensions’ development over time give an indication of what aspects of linguistic variation each captures.

**TABLE 1:** The five dimensions of variation in COHA.

Dimension label	Most salient features	Genre differentiation	Diachronic development
Structural elaboration	Clausal coordination, noun phrase trigrams, prepositions, main verb BE, attributive adjectives	Highest in nonfiction writing; lowest in magazines and newspapers	Consistent decrease in all genres
Verbal-personal communication	Pro-verb DO, private (cognitive) verbs, verbal infinitives, first person pronouns, adverbs	Highest in fiction writing, lower in all other genres	Increase in the non-fiction genres in the second half of the 20th century
Information density	Attributive adjectives, mean word length, type-token ratio, nouns, prepositions	Highest in nonfiction, lowest in fiction	Increase in all genres, particularly in the 20th century
Narration	Simple past, third person pronouns, possessives, quotation marks, public (quotative) verbs	Highest in fiction, lowest in nonfiction	Consistent increase in fiction; irregular developments in other genres
Abstraction & generalization	Prepositions, nominalizations, agentless passives, mean word length, infinitives	Highest in newspapers and nonfiction writing, lowest in fiction and magazines	Newspaper, nonfiction, and magazine writing grow closer to the consistently low values for fiction

Both the social and linguistic dissemination measures are based on discrete counts, which are not available for register as we operationalize it. A different method for quantifying register dissemination is therefore required than those used for social and linguistic dissemination above. Two options suggest themselves. First, similarly to Altmann et al. (2011) we can treat the presence or absence of *whom* in a text as a binary variable. For each step in the time period under analysis, we can then divide our corpus in two groups of texts, those including and not including *whom*. Both of these groups can be characterized as multivariate Gaussian distributions in the five-dimensional register-score space. Register dissemination can then be treated as the distinctiveness of the *whom*-texts from those without *whom* in register space. If there is significant overlap between both groups, this can be taken to indicate relatively wide dissemination, whereas if the groups are found to be largely distinct, this is a sign of register-specificity. The amount of overlap between two multivariate Gaussian distributions can be expressed as the Bhattacharyya distance (Bhattacharyya, 1943) between them.

This method is susceptible to differences in text length, since longer texts have a higher baseline probability of including a given feature and hence ending up in the *whom*-group. One solution would be to sub-divide larger texts into smaller segments to achieve uniform text length, and to treat each segment as a sample in its own right. While this would be a feasible solution in principle, a more plausible one is to treat relative frequency of *whom* in a text as a scalar variable. Doing so accounts for the effect of text length in a principled way without requiring further manipulation of the data. Instead of creating distinct groups, this method situates texts on a *whom*-frequency continuum.

In order to quantify the association between *whom* and specific register properties, we fit a linear model at each year, with relative frequency of *whom* as the outcome and each text’s scores for the five dimensions as the predictor variables. The adjusted *R* ^2^ values of these models are taken as indices of register specificity. A dissemination coefficient with similar properties to that proposed by Altmann et al. (2011) can then be obtained by subtracting this adjusted *R* ^2^ from 1. The more predictive power the joint dimension scores yield regarding relative frequency of *whom*, the higher the model’s *R* ^2^ value and the lower the corresponding D^R^. As with Altmann et al.’s (2011) index, a value of 1 indicates completely even dissemination in register space, whereas values below 1 suggest register clumping and consequently decreased vitality of the form.

In addition to the general D^R^, the values of each dimension’s model coefficients can also be traced over time, giving a sense of which register dimensions are most predictive of *whom*-frequency and which are most subject to change over time.

### Topic Dissemination

Apart from social and register properties, discourse topic may be an important predictor of linguistic variation. We create a topic model for COHA, which we restrict to 100,000 randomly selected texts for computational reasons. Specifically, we use latent Dirichlet allocation (LDA), which represents a predefined number of topics as probability distributions over the words in the corpus and treats every corpus text as a probability distribution over all topics (Blei et al., 2003).

Before generation of the topic model, the corpus data were preprocessed in the following manner: all words were lemmatized based on the information already included in COHA, and only words from the part-of-speech categories noun, verb, adjective, and adverb were retained. Sequences of proper nouns such as “United States” were treated as single words, once again drawing on the information provided in COHA. Finally, the top 1,000 bigrams and trigrams with a minimum absolute frequency of 100 were also treated as single units. Extraction and ranking of bi- and trigrams was done with NLTK’s collocations module (Bird et al., 2009), which uses pointwise mutual information as its association metric.

The LDA models themselves were constructed in Python’s (Python Software Foundation, 2020) gensim module (Řehůřek and Sojka, 2010), with the parameters chunksize set to 2,000, passes to 5, and iterations to 200. Such models were built for numbers of topics between 9 and 200. For each of these, model coherence was calculated with the *C*_*v*_ measure proposed in Röder et al. (2015). The candidate with the highest coherence is a 25-topic model. As with the register dimensions, it is not our aim here to discuss individual topics. Therefore, Table 2 simply shows the top five words in each topic to give a sense of the range and plausibility of the model on the whole.

**TABLE 2:** The 25 LDA topics developed for COHA.

Topic	Top words	Topic	Top words
1	candidate, democrat, kennedy, nixon, reporter	14	george, mexico, mexican, madeline, rollo
2	team, player, film, coach, movie	15	prince, queen, lord, rome, duke
3	boat, captain, sail, deck, crew	16	chinese, china, mountain, stone, surface
4	railroad, machine, steel, contract, profit	17	percent, budget, investment, oil, sales
5	soul, heaven, lord, dear, sir	18	peter, shot, int, sam, camera
6	p.-a., dear, aunt, mary, sir	19	sir, captain, colonel, horse, soldier
7	kid, guy, stare, phone, nod	20	patient, hospital, <br>, medical, drug
8	paul, bird, planet, flower, moon	21	senate, teacher, governor, amendment, candidate
9	cook, milk, fruit, sugar, meat	22	moral, religion, science, christian, religious
10	horse, wood, dog, mountain, stare	23	tom, joe, ben, ruth, phil
11	poet, poem, jane, poetry, novel	24	novel, magazine, editor, publisher, reader
12	animal, research, science, scientist, cell	25	governor, indian, lincoln, county, trial
13	soviet, russian, communist, germany, russia		

Our procedure of quantifying topic dissemination is largely the same as that for quantifying register dissemination, with one addition. The factor analytic procedure that produces register scores ensures that these are already uncorrelated, or only moderately correlated in the case of oblique rotation methods (Thompson, 2004). The opposite is the case for the topic probabilities of each text. Since these always sum to 1, they are fully collinear as a set and cannot be used directly as model predictors. We therefore subject them to principal component analysis and use the values of the principal components as predictors. This has disadvantages if one wishes to explore the effects of individual topics, but is entirely robust for evaluating the predictive power of the topic structure on the whole.

## Results

### Social Dissemination

**FIGURE 2:**
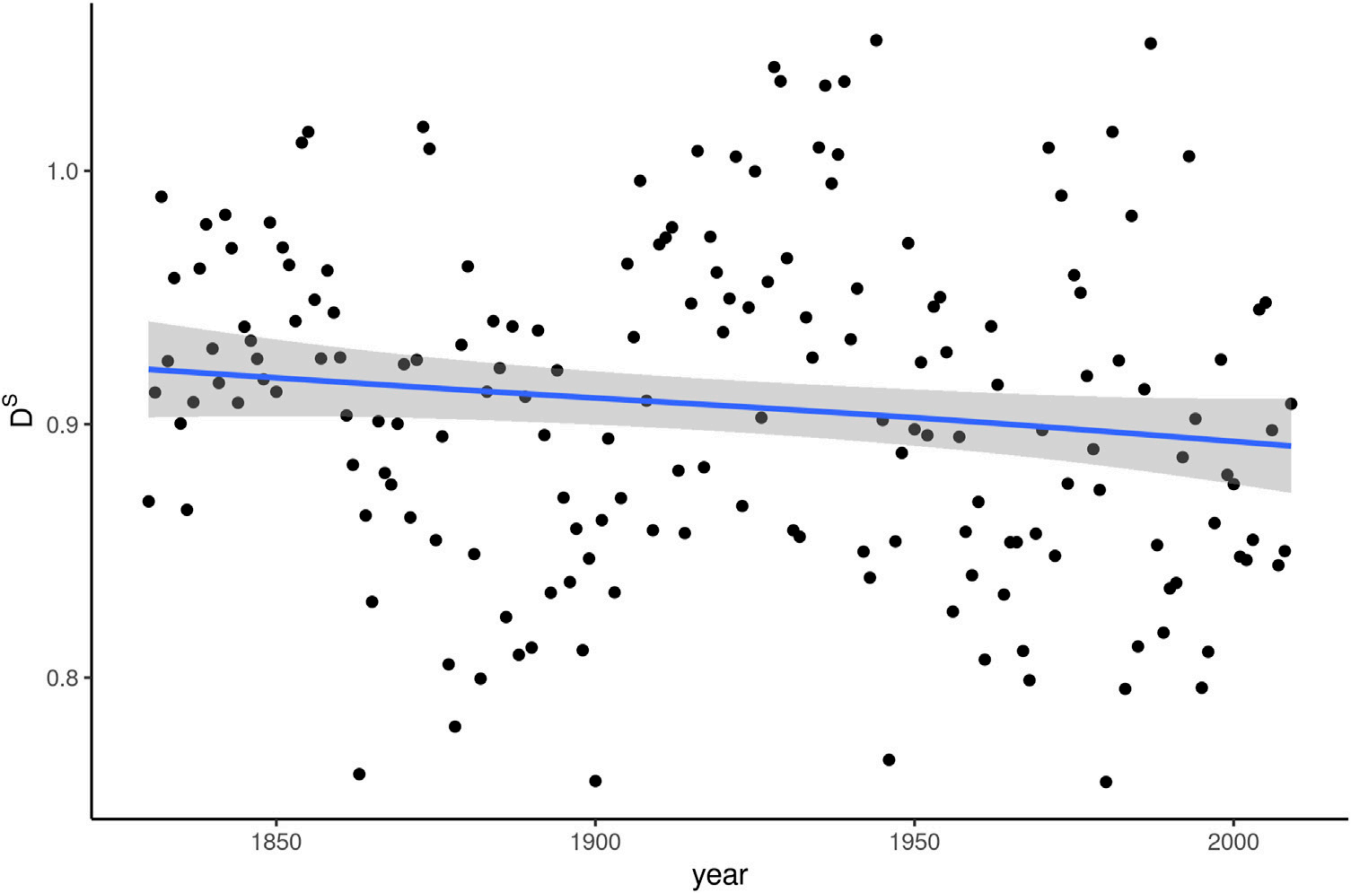
Social dissemination (D^S^) of *whom* over 180 years of written American English.

The development of social dissemination (D^S^) of *whom* between 1830 and 2009 is shown in Figure 2. In addition to plotting dissemination scores per year, the figure shows the curve of predicted values based on a generalized additive model with a cubic spline and four knots.^3^ The same curve formula is used for all plots below and was chosen to strike a compromise between being able to address nonlinear relationships in the data on the one hand and being relatively robust to noise on the other. In the case of social dissemination, the curve almost perfectly approaches a straight line, whose confidence intervals overlap throughout the interval covered by the data. The slight negative slope is therefore of little statistical consequence. The considerable spread of individual, yearly D^S^ values throughout the period analyzed further confirms this impression.

The results shown in Figure 2 are unspectacular on the whole. There is little information in this plot that sets *whom* apart from other words, either in terms of its general dissemination tendency or its dissemination dynamics over time. A mean social dissemination value of around 0.9 is entirely normal, since values below 1 are “in fact observed for most words” (Altmann et al., 2013: 3). *Whom* has a frequency of occurrence of about 2.5 * 10^–4^ in COHA. For words with similar frequency profiles, Altmann et al., (2011: 3) report median dissemination values around 0.8. The values in Figure 2, therefore, are above rather than below expected. Consequently, there is little indication that restricted dissemination accounts for the word’s frequency decline.

Looking at the development of social dissemination over time, the stability of the values in Figure 2 is moderately surprising. True enough, during the plotted time interval, *whom* continuously decreases in frequency. However, the decrease is not linear. It starts out relatively slowly, picks up speed in the second half of the 19th century and flattens out again after around 1950 (see Figure 1). Given that the relationship Altmann et al. (2011) and Altmann et al. (2013) establish is between change in frequency and change in dissemination, one would expect the bends in the frequency curve to coincide with changes in D^S^, yet this is not the case.

We further tested for the correlation between Δf and ΔD^S^, i.e. the change in both frequency and social dissemination measured in each year compared to the previous one. The result of a Pearson test did come out as significant (*p* < 0.001), but with a negative correlation of −0.261. This finding runs directly counter to expectations based on the attested relationship between frequency and social dissemination.

### Linguistic Dissemination

**FIGURE 3:**
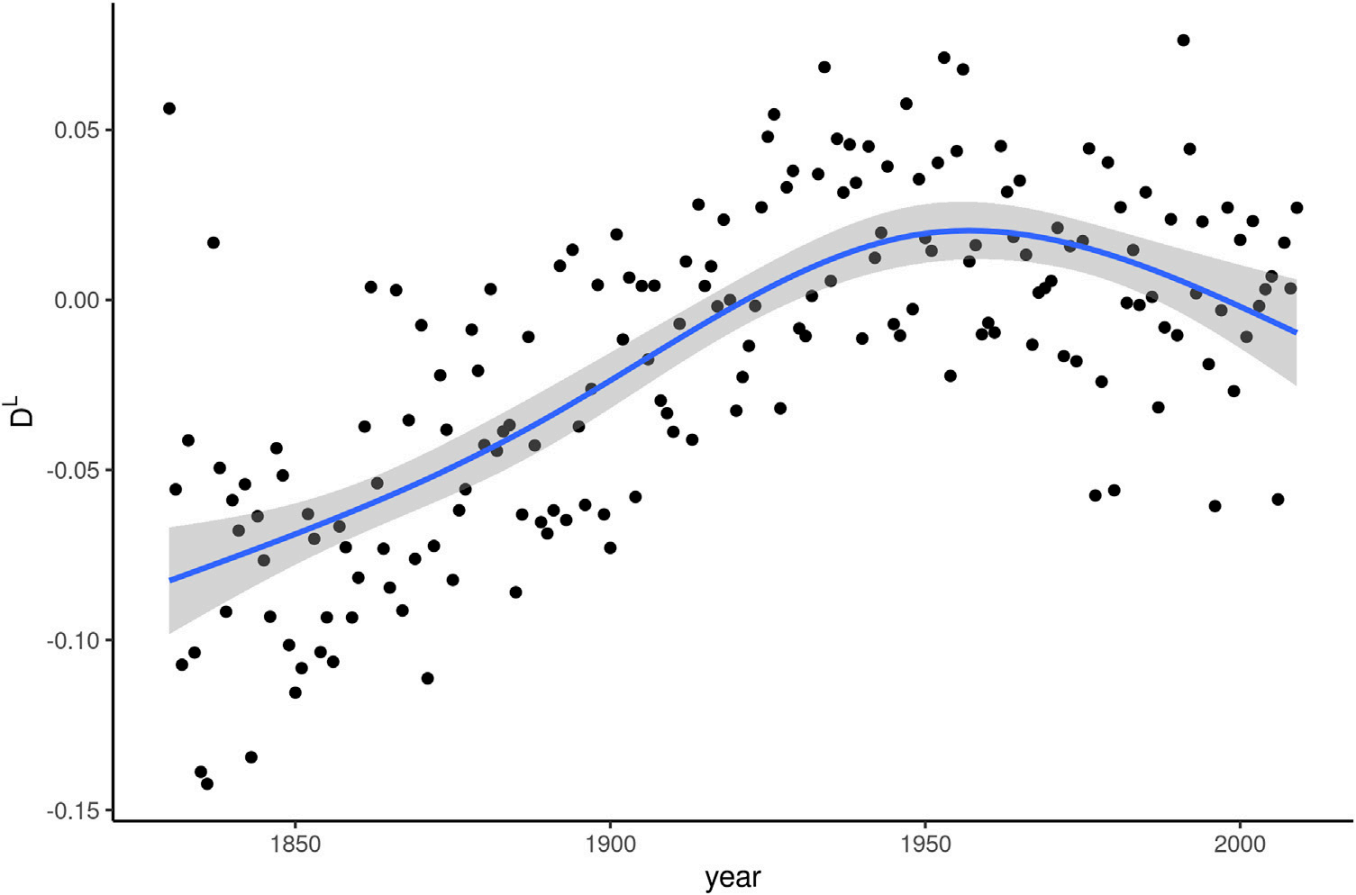
Linguistic dissemination (D^L^) of *whom* over 180 years of written American English.

Figure 3 shows the linguistic dissemination (D^L^) indices along with the smoothing curve in the same fashion as Figure 2. The difference between the two plots is immediately apparent. D^L^ appears to undergo a much more dynamic development than D^S^. The mean value of D^L^ for all words by definition is 0, with a standard deviation of 0.15 in our data. This means that *whom* starts out with linguistic dissemination values in the 19th century that are below average, but not strikingly so, as they are only about half a standard deviation from the mean. During the roughly 100 years of most intense frequency decline, between 1850 and 1950, linguistic dissemination actually increases steadily. In other words, while *whom* is receding in general, it appears to be gaining, not losing, linguistic versatility. In the second half of the 20th century, a time during which the frequency decrease slows down, D^L^ appears to reverse this upward trend.

The same correlation test as for the relationship between Δf and ΔD^S^ was also run for Δf and ΔD^L^, with similar results. A coefficient of −0.387 (*p* < 0.001) confirms the impression from Figure 3 that decline in frequency coincides with increase in linguistic dissemination.

In sum, linguistic dissemination develops almost entirely in the opposite direction from what might be expected based on the literature. Instead of a hypothesized positive correlation between D^L^ and frequency, extended periods of frequency decline coincide with a rise in D^L^ and periods of comparable frequency stability go hand in hand with a dip in linguistic dissemination.

### Register Dissemination

**FIGURE 4:**
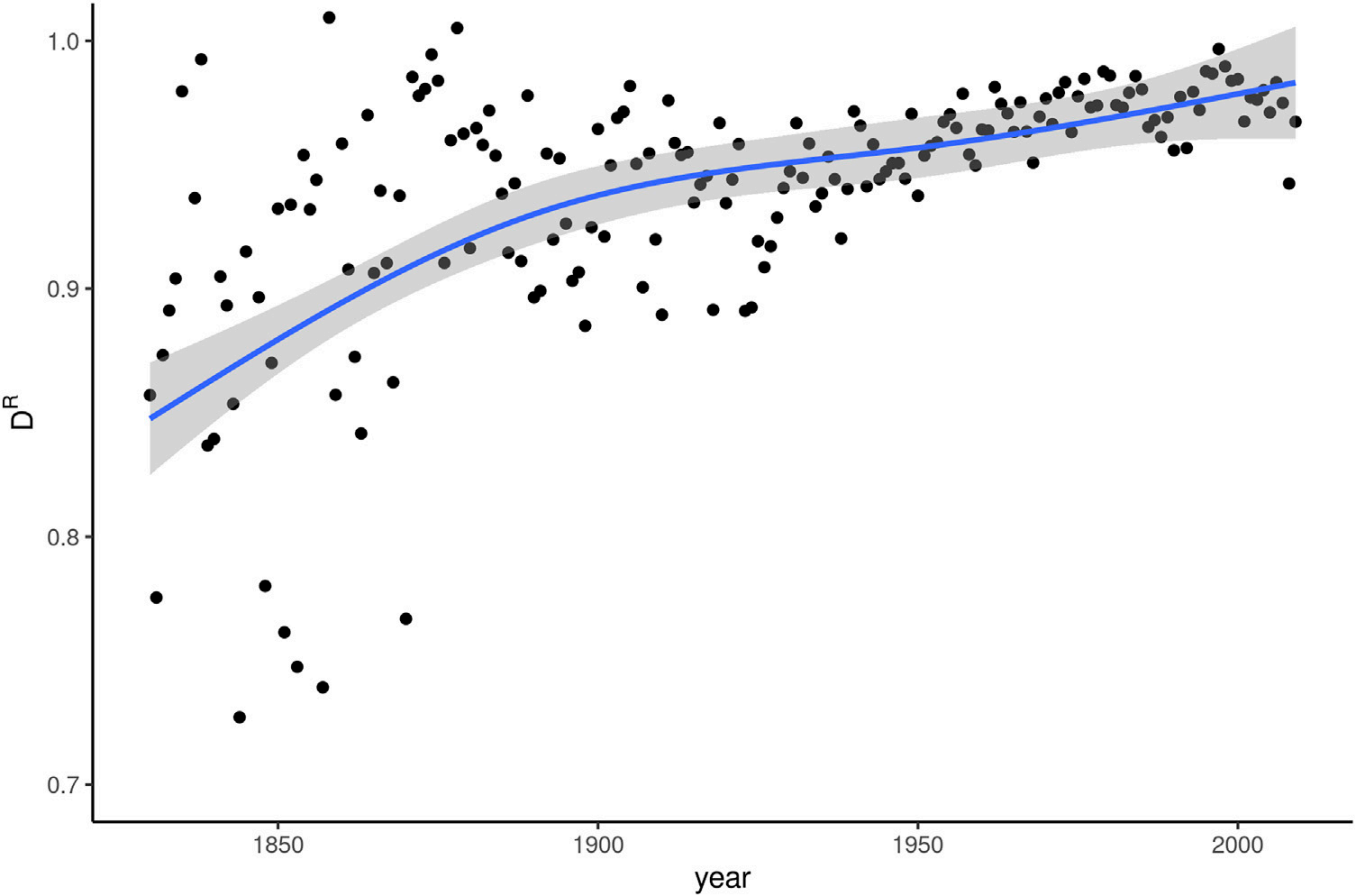
Register dissemination (D^R^) of *whom* over 180 years of written American English.

Figure 4 shows the D^R^ indices for linear models predicting relative frequency of *whom* based on the five dimension scores developed in the MDA described above. The curve of predicted values approaches a straight line with an upward slope, indicating that in earlier years, information about texts’ dimension scores is better able to account for variation in *whom*-usage.

With the exception of the earliest decades in the data, D^R^ is consistently above 0.8. Accordingly, register plays only a very limited role in predicting the frequency of *whom*. The smoothing curve approaches 1 for years after 2000. In the latest years of COHA, then, register information is almost entirely uninformative as to expected frequencies of *whom*. The conclusion has to be drawn that around the turn of the 21st century, there is almost no register differentiation left to characterize *whom* in actual usage. Once again, this pattern is directly opposite to expectations based on the known relationship between dissemination and frequency change for emerging words. A correlation test between Δf and ΔD^R^ produced no significant results. We assume that this is due to relatively large fluctuations in D^R^ for individual years and the comparatively longer time window over which register developments operate. Therefore, D^R^ may be better suited to address developments over more coarsely-grained time periods.

**FIGURE 5:**
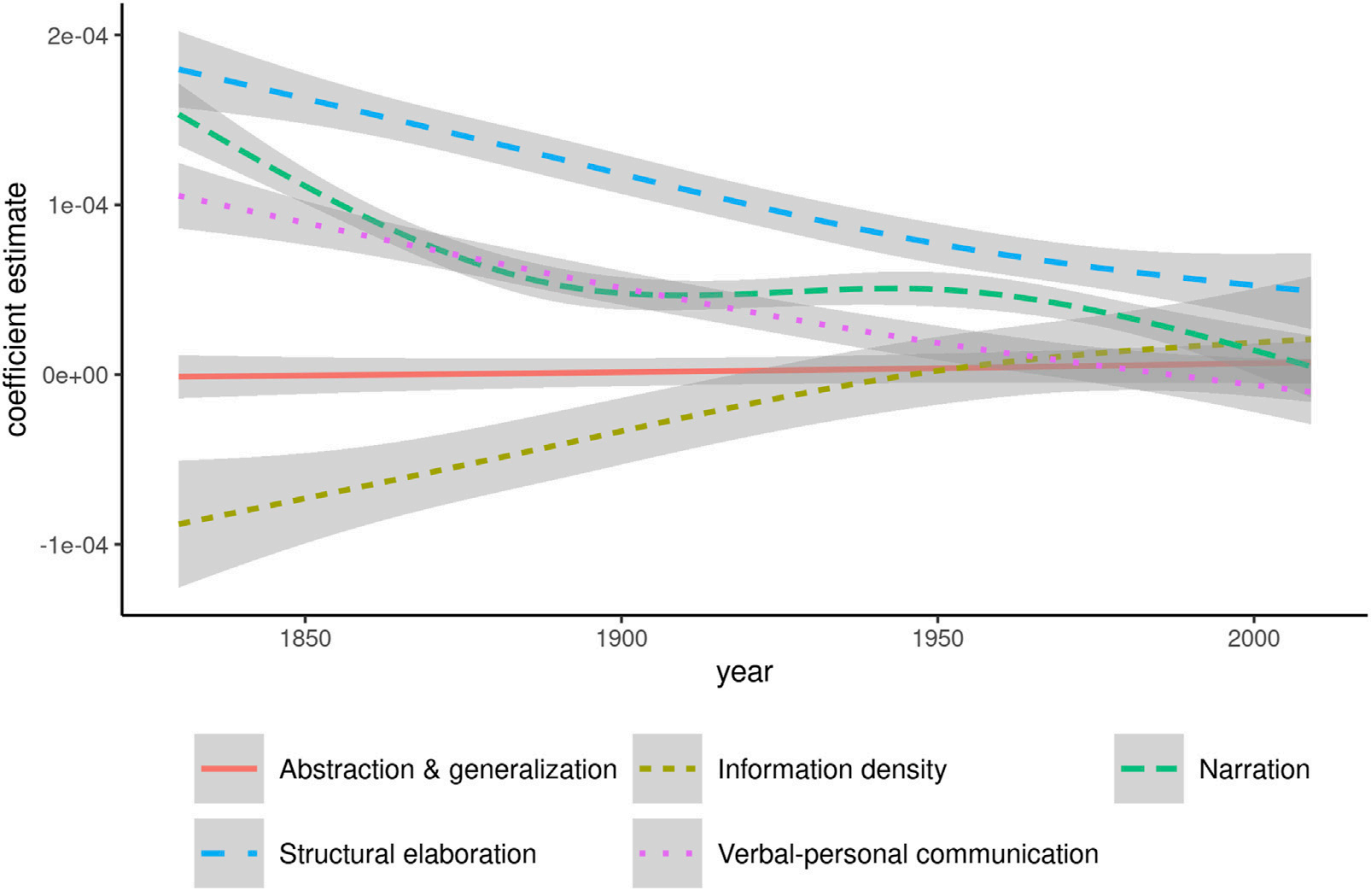
Coefficients for individual dimensions in models predicting frequency of *whom*. Points for individual years were omitted to avoid overplotting.

In relation to register, it is worth moving beyond the bird’s eye view of all dimensions in conjunction and to consider how individual dimensions relate to the general trend identified in Figure 4. To this purpose, Figure 5 shows smoothing curves of the coefficient estimates for each of the five dimensions calculated for each year.

Narration, Structural elaboration, and Verbal-personal communication all show a relatively steady regression from positive values towards 0. That is, an initial association between high text-scores along these dimensions and higher frequencies of *whom* decreases in strength in all three cases. The coefficients for Abstraction & generalization are indistinguishable from 0 throughout the entire period analyzed, showing that this dimension has no role to play in predicting the frequency of *whom*. Information density is the only dimension with an initially negative coefficient, which however increases steadily until it intersects 0 around 1950. From this point on, the coefficient values are positive, but so low that they are effectively indistinguishable from 0. This pattern suggests that *whom* is associated with comparatively loose packaging of information throughout the 19th century, but becomes increasingly associated with information density in the 20th century.

At a more general level, the convergence towards 0, i.e. no measurable effect, for all dimensions is a striking pattern. By the 2000s, the only coefficient that is appreciably different from 0 is that for Structural elaboration, and even this estimate is reduced to about half its value compared to the 1830s. Figure 5 draws a much more vivid picture of the increasing register dissemination of *whom* throughout the period under analysis, a process that is almost complete by the last years covered in COHA.

### Topic Dissemination

**FIGURE 6:**
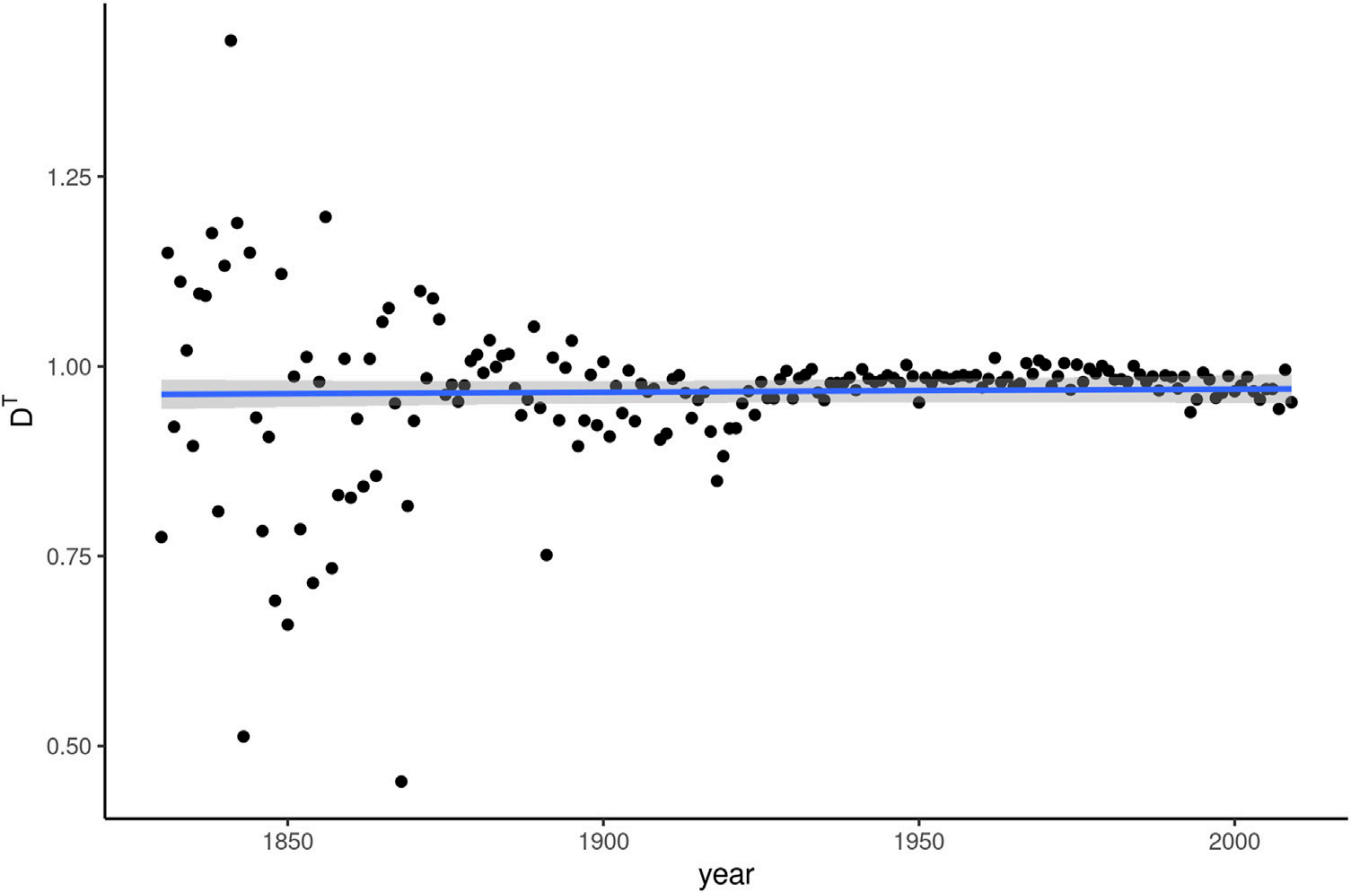
Topic dissemination (D^T^) of *whom* over 180 years of written American English.

The story of topic dissemination is quickly told: no significant effect of discourse topic on *whom* can be discerned. This is readily apparent from Figure 6, which shows the D^T^ values derived from linear models predicting *whom* frequency based on (rotated) topic distributions in the texts. The values are very close to and at times even above 1. The latter is due to the models’ *R* ^2^ being adjusted downward for predictor variables that add more complexity than predictive power, as is likely the case with some components derived from principal component analysis. More importantly, the minimal fluctuation in R^2^ over time is not sufficient to indicate any diachronic pattern.

## Discussion

### Dynamics of Emerging Versus Receding Forms

Our results do not confirm any of the expectations one might derive from the extant literature on the relationship between word growth/decline and dissemination. The decrease in frequency of *whom* does not coincide with systematic decrease in any of the dissemination measures, nor do the individual measures themselves correlate to draw a unified picture. Pairwise Pearson tests between the four measures reveal one relatively weak correlation of note: linguistic and register dissemination show a Pearson’s coefficient of 0.398 at *p* < 0.001. All other correlations do not reach statistical significance even at the least conservative conventional level of *p* < 0.05.

At least for the particular case of *whom*, then, there is little evidence to suggest that dissemination dynamics during the decline of an established feature parallel those during the emergence of innovative words. Results for a larger number of receding features are required to further substantiate the nature and degree of the differences suggested by the results above. We have explored the dissemination measures used here for the 200 most rapidly receding word forms in COHA and found item-specific differences to be more noticeable than any unified trend. The general statement can certainly be made, however, that there is no pervasive trend in the expected direction of decrease in dissemination correlating with decrease in frequency. Future work will have to address more fully whether meaningful statistical generalizations can be made about dissemination dynamics of receding words. However, as we explain in *Research Objectives*, we believe that item-specific explanations beyond general statistical tendencies are necessary for a sociolinguistically accountable discussion.

### Contextualization Against the Sociolinguistic Record

From a sociolinguistic perspective, we do not necessarily consider the above findings alarming. The relationship between dissemination and frequency change is a statistical tendency that has been shown to hold as a generalization over large numbers of emerging words. Yet the dynamics of individual words are governed by more than the global statistical properties identified in Altmann et al. (2011), Altmann et al. (2013), or Stewart and Eisenstein (2018). In the case of *whom*, we have access to item-specific explanatory factors, such as the erosion of the English case system and the prolific metalinguistic discourse surrounding the word. As such, we can relate the change observable in the dissemination measures to this information in order to more fully understand the pathway of *whom* over the past 200 years. In this perspective, the dissemination measures are recontextualized as heuristic tools rather than variables used to test generalized hypotheses.

Returning to the extant literature on *whom*, a sketch of three developmental stages over the past three centuries can be drawn that is both in line with findings from previous research and able to account for dissemination developments as presented in our results. This sketch sees *whom* develop from 1) a carrier of grammatical information that is categorically required in a well-defined number of linguistic contexts to 2) a sociolinguistic variable that increasingly acquires stylistic over grammatical constraints and, finally, 3) a vestigial element which hardly shows productive variation in usage but retains salience thanks to active metalinguistic debate.

According to Aarts (1994), grammarians of the 18th and early 19th century treated variation between *who* and *whom* as a clear-cut case of complementary distribution: The former was reserved as the subject relativizer in RCs with human antecedent, whereas the latter was required both for direct objects and for complements of prepositions. That prescriptivist authors felt the need to formulate such a rule hints at the fact that there was some variation even in the 18th century, but at the same time the precept “became one of the most popular prescriptive rules in English grammar” (Aarts, 1994: 73). Its continued sway until well into the 20th century can be inferred from Sapir (1921: 166–174), who expresses unease at diverging from the normative pattern while at the same time recognizing the clear drift of English grammar away from *whom*.

During this first idealized stage, while grammatical context provides an unambiguous criterion for the choice between *who* and *whom*, the latter enjoys relative safety from the factors conspiring to ultimately lead to its demise: the overwhelming loss of nominal case inflection and the encroachment of other relativizers into its territory. However, the categorical, purely grammatical rule gradually morphed into a more context-sensitive one. Aspects of style were taken into account alongside, and increasingly: above, questions of case agreement. When and how precisely this change occurred has not yet been fully documented; Aarts (1994: 73) cites examples from 1985 onwards, leaving a gap of roughly 150 years to the most recent example of the former, rigid grammatical rule (Cobbett’s *A Grammar of the English Language* from 1818).

Irrespective of the precise chronology, which can be assumed to have taken a gradual development at any rate, the relaxing of strict grammatical constraints made *whom* available as an indexically marked choice. A look at our data, and specifically: the development of register dissemination in Figures 4 and 5, suggests that the relaxing of the rule must have been in operation in the early 19th century already. At this time, *whom* is associated with elaborate and verbose texts, as can be inferred from the positive coefficients for the dimension Structural elaboration and the negative ones for Information density. In other words, stylistic constraints had already come to play an important role around 1830.

In the long run, these associations likely did not help *whom* to retain much of its vitality. Throughout the recent history of English, there have been pervasive trends towards more efficient packaging of information and structural simplicity (Leech et al., 2009). As such, the rapid frequency decrease *whom* experiences between around 1850 and 1950 appears plausible. What is more puzzling at first glance is the concomitant increase in both linguistic and register dissemination. As to the former, the weakening of strict grammatical conditioning offers an explanation. While generally becoming less frequent, the occurrence of *whom* can no longer be predicted entirely from its immediate syntactic context. This is the case for RCs that formerly would have allowed no alternative to *whom*, but start to increasingly occur with *who.* Yet, perhaps more important are constructions in which *whom* would not have been permissible previously, but where hypercorrect application of an increasingly intransparent grammatical rule leads to its occasional, erratic appearance. Such hypercorrect usage is attested, among others, in Sledd (1987) and Tabbert (1990).

The increasing register dissemination visible throughout the period we analyze (see Figure 4) is an early sign of the last stage in our schematic representation, the retreat of *whom* from the vernacular grammar of most native speakers. Combined with the continued frequency decrease, the even register dissemination by the latter half of the 20th century suggests that *whom* simply is hardly used anymore at all, regardless of particular stylistic properties of individual texts. We observe its use mainly in two kinds of context: first, a small set of grammatical constructions that offer no easy alternative to replace *whom* by *who*, such as (2), and second, metalinguistic instances where *whom* is the subject of discourse rather than simply part of the discourse itself. Examples like the *who–whom–whomst–whomst’d* meme cited above, or a recent book entitled *A World Without* Whom*: The Essential Guide to Language in the BuzzFeed Age* (Favilla, 2017), highlight this latter usage.

(2) The characters, **between whom** the distances are long and harsh <COHA_mag_1989_486754>

Our interpretation that, by the late 20th century, *whom* is no longer a productive element in the active competence of most native speakers of English is in line with Bauer’s (1994: 77) observation that readers in the late 20th century will generally recognize correct use of *whom* in a far wider range of contexts than they actually use it. It also finds confirmation in Mair (2006: 143) assessment that “*whom* is moribund as an element of the core grammar of English, but is very much alive as a style marker whose correct use is acquired in the educational system.” Lasnik and Sobin’s (2000) proposal to treat *whom* as a “grammatical virus” extraneous to vernacular grammar also hits a similar line.

This sketch of the historical development *whom* has undergone over the past 200 years, then, is able to accommodate the linguistic and register dissemination developments identified in our results. We summarize these relationships in Table 3. The role of social and topic dissemination remains less clear, partly because neither measure appears to correlate with frequency developments of *whom* in any meaningful way. It is possible that discourse topic simply has little bearing on the choice of relativizer, but we would expect social dissemination to yield clearer results, at least at times during which *whom* starts to acquire stylistic meanings. The most likely explanation for the absence of any clearer findings, we believe, lies in the nature of the data. With relatively few texts per year, especially in the earlier half of COHA, estimates of social dissemination suffer from considerable noise. The spread of individual points in Figure 2 is a sign of this problem. We would expect a larger corpus database to offer clearer results.

**TABLE 3:** Three developmental stages of whom.

Period	Status of *whom*	Sociolinguistic effects	Dissemination developments
Prior to 19th century	Regular grammatical conditioning	Stability due to categorical rules for relativizer choice	Not covered by our data; hypothesized stability of dissemination
19th and early 20th century	Predominantly stylistic conditioning	Variability between *who* and *whom; who* encroaches upon traditional *whom* contexts; hypercorrect use of *whom* due to intransparent grammatical rule	Low register dissemination indicating stylistic specificity; increase in linguistic dissemination as a consequence of weakening grammatical conditioning
1950s onward	Retreat from active use	Avoidance of *whom*; active metalinguistic discussion; discrepancy between awareness and use	Even dissemination due to overall low frequencies in all contexts

### Conclusion and Outlook

We have tested the association between frequency developments and changes in a range of word dissemination measures in the case of one receding word, *whom,* on the basis of historical corpus data comprising 180 years of written American English. In addition to the established metrics social dissemination (Altmann et al., 2011; Altmann et al., 2013) and linguistic dissemination (Stewart and Eisenstein, 2018), we have introduced two novel measures to quantify the dissemination of a word across registers and topics. The significant positive correlation between frequency and dissemination attested in the literature on emerging words was not found to hold for receding features.

Of the four measures we have considered, only linguistic and register dissemination showed meaningful changes between 1830 and 2009. These proved difficult to interpret in terms of general statistical tendencies, but became plausible once the specific sociolinguistic history of *whom* was considered. We proposed a trajectory of development, according to which *whom* changed from a regular grammatical to a predominantly stylistic marker and, in the latter half of the 20th century, to an unproductive element whose salience far supersedes its actual use.

Following Squires (2014), we submit that the relationship between dissemination and word vitality is best not conceived as static, but may assume different shapes at different stages of development. A productive goal for future research will be to reconcile this flexibility with an analytical perspective that goes beyond isolated, contextualized words. We are currently exploring unsupervised learning methods to find natural groups of words, based on their frequency and dissemination profiles across time. For instance, among the 50 most rapidly receding surface forms in COHA, we find some linearly declining social dissemination developments (e.g. for *nor* and *shall*), some random fluctuations around relatively constant values as with *whom* (e.g. also for *borne* and *circumstance*) as well as more complex, curvilinear trajectories (e.g. for *till* or *subject*). Based on a matrix of frequency information as well as (social, linguistic, register, and topic) dissemination at different intervals for individual words, we are working towards clustering words into groups that show similar developments over time.

The question remains why social and topic dissemination appear stable throughout the dynamic development of *whom* sketched above. D^T^ may simply not play an important role in general. The effect of discourse topic on linguistic variation is currently not well understood, as sociolinguists have often preferred to focus their analyses on different ways of saying the same thing (Labov, 1972: 271) rather than differences in what people talk about. More active consideration of discourse topic as a predictor of variation in sociolinguistics in general would be necessary to better interpret our findings in relation to topic dissemination.

As for social dissemination, it is difficult to accept that no notable change occurs alongside the decline of *whom* between 1830 and 2009. We have argued above that the measure may yield unstable results if the amount of individual texts is not sufficiently large, as reflected in the wide spread of yearly D^S^ values in Figure 2. Unfortunately, this property makes the measure problematic for many sociolinguistic applications, for which often only relatively small corpora are available. By contrast linguistic dissemination is able to draw on information from every instance of a feature’s use and our new metric of register dissemination uses fine-grained, scalar information at the level of individual texts. Consequently, we expect both these measures to be better suited for application to comparatively small data sets.

## Data Availability

Publicly available datasets were analyzed in this study. This data can be found here: https://www.english-corpora.org/coha/ for online access to the corpus itself https://github.com/axboh/WHOM_Dissemination for all intermediate datasets created for the analyses presented in the paper.

## Footnotes

1 See https://medium.com/write-i-must/dank-etymology-the-middle-english-origins-of-whomst-374ecd7a96fa for examples and an extended journalistic discussion.

2 See https://www.english-corpora.org/coha/

3 To fit this curve, the following command was added to the plot objects in R:geom_smooth(method=”gam”, formula = y ∼ s(x, bs = “cs”, k = 4)).

## References

[B1] Aarts F. Aarts B. (2002). “Relative *Whom*: ‘A Mischief maker.’,” in Text Types And Corpora: Studies in Honor of Udo Fries. Editors Fischer A. Tottie G. Lehmann H. M. (Tübingen, Germany: Narr), 123–130.

[B2] Aarts F. (1994). Relative *Who* and *Whom*: Prescriptive Rules and Linguistic Reality. Am. Speech 69 (1), 71. 10.2307/455950

[B3] Altmann E. G. Pierrehumbert J. B. Motter A. E. (2011). Niche as a Determinant of Word Fate in Online Groups. PLoS One 6 (5), e19009. 10.1371/journal.pone.0019009 21589910 PMC3093376

[B4] Altmann E. G. Whichard Z. L. MotterMotter A. E. (2013). Identifying Trends in Word Frequency Dynamics. J. Stat. Phys. 151 (1–2), 277–288. 10.1007/s10955-013-0699-7

[B5] Bauer L. (1994). Watching English Change: An Introduction To the Study of Linguistic Change in Standard Englishes in the Twentieth Century (Learning About Language). Harlow, United Kingdom: Longman.

[B6] Bhattacharyya A. K. (1943). On a Measure of Divergence Between Two Statistical Populations Defined by Their Probability Distributions. Bull. Calcutta Math. Soc. 35, 99–109.

[B7] Biber D. Conrad S. (2009). Register, Genre, and Style. Cambridge, United Kingdom: Cambridge University Press.

[B8] Biber D. (1988). Variation across Speech and Writing. Cambridge, NY: Cambridge University Press.

[B9] Biber D. (2012). Register as a Predictor of Linguistic Variation. Corp. Linguist. Linguist. Theor. 8 (1), 9–37. 10.1515/cllt-2012-0002

[B10] Bird S. Klein E. Loper E. (2009). Natural Language Processing with Python: Analyzing Text With the Natural Language Toolkit. Beijing, Cambridge, MA: O’Reilly Media.

[B11] Blei D. Ng A. Y. Jordan M. I. (2003). Latent Dirichlet Allocation. J. Mach. Learn. Res. 3, 993–1022. 10.1162/jmlr.2003.3.4-5.993

[B12] Bohmann A. (2019). Variation in English Worldwide: Registers and Global Varieties (Studies in English Language). Cambridge, United Kingdom: Cambridge University Press.

[B13] Davies M. (2010). Corpus of Historical American English (COHA). Available at: https://www.english-corpora.org/coha/ (Accessed December 20, 2020).

[B14] Egghe L. (2007). Untangling Herdan’s Law and Heaps’ Law: Mathematical and Informetric Arguments. J. Am. Soc. Inf. Sci. 58 (5), 702–709. 10.1002/asi.20524

[B15] Favilla E. J. (2017). A World without “Whom”: The Essential Guide to Language in the BuzzFeed Age. New York: Bloomsbury.

[B16] Garley M. Hockenmaier J. (2012). “Beefmoves: Dissemination, Diversity, and Dynamics of English Borrowings in a German Hip Hop Forum,” in Proceedings of the Association of Computational Linguistics, Jeju Island, Korea, July 2012 (Jeju Island, Korea: Association for Computational Linguistics), 135–139.

[B17] Grieve J. (2018). “Natural Selection in the Modern English Lexicon,” in Proceedings of the 12th International Conference on the Evolution of Language (Evolang12), Torun, Poland, April 17, 2018 (Wydawnictwo Naukowe Uniwersytetu Mikołaja Kopernika).

[B18] Grieve J. Nini A. Guo D. (2017). Analyzing Lexical Emergence in Modern American English Online. English Lang. Linguist. 21 (1), 99–127. 10.1017/S1360674316000113

[B19] Guy G. R. Bayley. R. (1995). On the Choice of Relative Pronouns in English. Am. Speech 70 (2), 148–162. 10.2307/455813

[B20] Hinrichs L. Szmrecsanyi B. Bohmann A. (2015). *Which*-hunting and the Standard English Relative Clause. Language 91 (4), 806–836. 10.1353/lan.2015.0062

[B21] Kretzschmar W. A. (2015). Language and Complex Systems. Cambridge, United Kingdom: Cambridge University Press.

[B22] Labov W. (1994). Principles of Linguistic Change (Vol. 1: Internal Factors) (Language in Society 20). Cambridge, MA: Blackwell.

[B23] Labov W. (1972). Sociolinguistic Patterns. Philadelphia, PA: University of Pennsylvania Press.

[B24] Lasnik H. Sobin N. (2000). The *Who/Whom* Puzzle: On the Preservation of an Archaic Feature. Nat. Lang. Linguist. Theor. 18 (2), 343–371. 10.1023/A:1006322600501

[B25] Leech G. N. Hundt M. Mair C. Smith N. (2009). Change in Contemporary English: A Grammatical Study (Studies in English Language). Cambridge, NY: Cambridge University Press.

[B26] Levey S. (2006). Visiting London Relatives. English World-Wide 27 (1), 45–70. 10.1075/eww.27.1.04lev

[B27] Mair C. (2006). *Twentieth Century English: History, Variation and Standardization* (Studies in English Language). Cambridge, United Kingdom: Cambridge University Press.

[B28] Python Software Foundation (2020). Python. Available at: http://www.python.org. (Accessed December 20, 2020).

[B29] R Core Team (2020). R: A Language and Environment for Statistical Computing. Vienna, Austria: R Foundation for Statistical Computing Available at: https://www.R-project-org/ (Accessed December 20, 2020).

[B30] Řehůřek R. Sojka P. (2010). “Software Framework for Topic Modelling with Large Corpora,” in Proceedings of the LREC 2010 Workshop on New Challenges for NLP, Valetta, Malta, May 22, 2010, 46–50.

[B31] Revelle W. (2020). psych: Procedures for Psychological, Psychometric, and Personality Research. Available at: https://CRAN.R-project.org/package=psych (Accessed December 20, 2020).

[B32] Röder M. Both A. Hinneburg A. (2015). “Exploring the Space of Topic Coherence Measures,” in Proceedings of the Eighth ACM International Conference on Web Search and Data Mining, Shanghai, China, February 5, 2015 (Shanghai: ACM), 399–408.

[B33] Sapir E. (1921). Language. London, United Kingdom: Harvest.

[B34] Sledd J. (1987). The Whoming Pigeon. Am. Speech 62 (4), 379–380. 10.2307/455417

[B35] Squires L. (2014). From TV Personality to Fans and Beyond: Indexical Bleaching and the Diffusion of a media Innovation. J. Linguist. Anthropol. 24 (1), 42–62. 10.1111/jola.12036

[B36] Stewart I. Eisenstein J. (2018). “Making “Fetch” Happen: The Influence of Social and Linguistic Context on Nonstandard Word Growth and Decline,” in Proceedings of the 2018 Conference on Empirical Methods in Natural Language Processing, Brussels, Belgium, November 4, 2018 (Brussels, Belgium: Association for Computational Linguistics), 4360–4370.

[B37] Tabbert R. (1990). Rare Whoming Pigeon Sighted in the grove of Academe. Am. Speech 65 (2), 164–165. 10.2307/455536

[B38] Thompson B. (2004). Exploratory and Confirmatory Factor Analysis: Understanding Concepts and Applications. Washington, DC: American Psychological Association.

